# Evaluation of physicochemical and antioxidant properties of two stingless bee honeys: a comparison with *Apis mellifera* honey from Nsukka, Nigeria

**DOI:** 10.1186/s13104-017-2884-2

**Published:** 2017-11-06

**Authors:** Justus Amuche Nweze, J. I. Okafor, Emeka I. Nweze, Julius Eyiuche Nweze

**Affiliations:** Department of Microbiology, Faculty of Biological Sciences, University of Nigeria, Nsukka, 41001 Nigeria

**Keywords:** Stingless, Honey, Physicochemical, Antioxidants, HMF, Phenolics, Flavonoids, Ascorbic acid, FRAP, AEAC

## Abstract

**Objective:**

Several physical, biochemical and antioxidant properties of two Nigerian stingless bee honey varieties (*Melipona* sp. and *Hypotrigona* sp.) were compared with *Apis mellifera* honey using standard analytical procedures.

**Results:**

The mean pH of *Apis mellifera, Hypotrigona* sp. and *Melipona* sp. honeys were 4.24 ± 0.28, 3.75 ± 0.11 and 4.21 ± 0.37 respectively. The mean moisture contents of the honeys were 11.74 ± 0.47, 17.50 ± 0.80, and 13.86 ± 1.06%. Honey samples from *Hypotrigona* sp. when compared with other honey samples had the highest mean total dissolved solids (370.01 ± 22.51 ppm), hydroxymethylfurfural (16.58 ± 0.37 mg/kg), total acidity (35.57 ± 0.42 meq/kg), protein content (16.58 ± 0.37 g/kg), phenol content (527.41 ± 3.60 mg/kg), and ascorbic acid (161.69 ± 6.70 mg/kg), antioxidant equivalent—ascorbic acid assay value (342.33 ± 0.78 mg/kg) as well as ferric reducing power (666.88 ± 1.73 μM Fe(II)/100 g) (p < 0.05). Several strong correlations were observed among some of the parameters of the honeys. This is the first study to compare the properties of Nigerian honey bees. Our results suggested that these honeys (specifically *Hypotrigona* sp. honey) is a good source of antioxidants comparable to *A. mellifera* honey.

**Electronic supplementary material:**

The online version of this article (10.1186/s13104-017-2884-2) contains supplementary material, which is available to authorized users.

## Introduction

Honey is a natural sweet sticky and viscous solution produced by honey insects from the nectar of flowers or from living parts of plants. The nectar is transformed into honey by honey bees and stored in the pot-like comb or waxy honeycomb inside the hive to ripen and mature for further use. Honey is a mixture of substances composed of mainly sugars and water, as well other compounds (less than 1%) such as proteins, minerals, enzymes, vitamins, 5-hydroxymethylfurfural (HMF), volatile compounds, flavonoids, and phenolic acids [1].

Even though honey is produced worldwide, its composition and antimicrobial activity can be variable, and are dependent primarily on their botanical origin, geographical and entomological source [2]. Other certain external factors, such as harvesting season, environmental factors, processing and storage condition, also play important roles [3]. Honey contains significant antioxidant compounds including ascorbic acid, flavonoids, glucose oxidase, amino acids, proteins phenolic acids, organic acids, carotenoid derivatives, and maillard reaction products [1, 3]. The biological properties that make it ideal as a medicine are: antibacterial, bacteriostatic, anti-inflammatory, wound and sunburn healing effects, antioxidant activity, radical scavenging activity and antimicrobial activity [3, 4].

Previous studies have reported some of the physico-chemical properties of Nigerian honey from *Apis mellifera* [5–9] and stingless bee [10] but to our knowledge there is scanty or no report on antioxidant properties of honeys from stingless bees usually found in Nsukka, Nigeria. Therefore, the aim of this study was to evaluate and compare the physico-chemical and antioxidant properties of three varieties of honeys from *A. mellifera*, *Hypotrigona* sp. and *Melipona* sp.

## Main text

### Materials and methods

#### Collection of honey samples

Nine multi-floral honey samples, two each from *Apis mellifera, Hypotrigona* sp. (locally called Okotobo in Igbo, Nsukka, Nigeria) and *Melipona* sp. (Ifufu or Ihuhu) were collected from different bee-keepers at Igbo Eze North Local Government Area (Enugu Ezike), in Enugu State (Nigeria) between June and July, 2015. The honey samples were harvested and kept at 5 °C.

#### Physicochemical analyses

The pH of a 10% (w/v) solution was measured according to Association of Official Analytical Chemists methods [11]. The moisture content was determined based on the refractometric method of International Honey Commission [2]. A 20% (w/v) solution of honey was prepared and using conductivity meter, electrical conductivity (EC) and total dissolved solids (TDS) of each sample were measured [12]. The colours of the honey samples were determined by spectrophotometric method according to the method of White [13] and the colour intensities (ABS_450_) were determined using the method of Beretta et al. [14].

#### Biochemical analyses

The refractometric technique was used to determine the total sugar contents (TSC) of a 25% (w/v) solution of honey [15] and using glucose standard solutions (100–600 μg/mL), the reducing sugar content was measured according to Saxena et al. [16] and the amount of sucrose content (%) was measured [15]. A technique based on the method described by Winkler was used to determine the HMF content of the samples [17] and it was calculated (mg/kg) according to Auerbach and Borries [18]. Using Lowry’s method [19], the protein content of honey was determined while the free acidities and lactones contents were determined by equivalence point titration according to IHC [2].

#### Analysis of antioxidant potentials

By using spectrophotometric Folin–Ciocalteu method [20], total phenolic content was determined based on gallic acid standard solutions (20–100 μg/mL; y = 0.0017x − 0.0104). The colorimetric assay used by Zhishen et al. was used to measure the flavonoid content [21] using a standard solution of catechin (20–100 μg/mL; y = 0.0136x − 0.0092). A method established by the IHC was used to estimate proline content [2] and the ascorbic acid content was determined based on the method described by Ferreira et al. [22], using a standard solution of pure l-ascorbic acid (50–400 µg/mL; y = 3.2460x − 0.0614). The antioxidant content was determined by measuring antioxidant equivalent ascorbic acid content (AEAC) values using the method described by Meda et al. [23] and a standard solution of pure l-ascorbic acid (1–8 µg/mL; y = 0.2746x − 0.0943). The ferric reducing/antioxidant power (FRAP) assay (μMFe(II)) was determined using the procedure described by Bertoncelj et al. [24].

### Statistical analyses

Results were reported as the mean ± standard deviation of duplicate experiments. Using SPSS statistical package (version 23), ANOVA and post hoc multi-comparison test were used for comparison of means (*p* < 0.05). Several parameters were correlated using Pearson’s correlation coefficient (r) (*p* < 0.01).

## Results

All the honey samples tested were acidic and the pH values of the honey samples varied (Table 1). There were significant differences between the mean values of pH (F_(2,24)_ = 30.99), TDS (F_(2,24)_ = 154.591), EC (F_(2,24)_ = 20.908), TDS (F_(2,24)_ = 154.591) and ABS_450_ (F_(2,15)_ = 169.73) (*p* < 0.05) of the honey varieties. *Hypotrigona* sp. honey had significantly the lowest mean pH value (3.75 ± 0.105, *p* < 0.05). A significantly highest mean values of moisture contents (17.50 ± 0.80%), EC (0.303 ± 0.04 mS/cm), and TDS (370.01 ± 22.51 ppm) were found in *Hypotrigona* sp. honey (*p* < 0.05) (Table 1). *A. mellifera* and the other two honey varieties were amber and light amber in colour (Pfund scale table), respectively.

**Table 1:** Physico-chemical parameters (pH, moisture, EC, TDS, colour and colour intensity) of various honey varieties

Honey source	Samples	Parameters					
		pH	Moisture (%)	EC (mS/cm)	TDS (ppm)	Colour (nm)	ABS_450_ (mAU,50w/v)
*Apis mellifera*	I	4.46 ± 0.095	11.870 ± 0.703	0.163 ± 0.0153	264.1 ± 0.59	1.986 ± 0.001	961.33 ± 0.58
	II	4.22 ± 0.077	11.537 ± 0.454	0.217 ± 0.1155	282.0 ± 0.25	2.102 ± 0.001	988.33 ± 1.15
	III	4.04 ± 0.124	11.817 ± 0.301	0.240 ± 0.0200	316.3 ± 0.06	2.108 ± 0.001	1006.33 ± 1.53
Mean		*4.24* ± *0.20* ^*b*^	*11.74* ± *0.47* ^*a*^	*0.207* ± *0.04* ^*a*^	*287.46* ± *22.95* ^*b*^	*2.065* ± *0.06* ^*b*^	*985.33* ± *19.64* ^*c*^
*Hypotrigona* sp.	I	3.85 ± 0.111	18.310 ± 0.900	0.317 ± 0.0058	399.1 ± 0.35	2.103 ± 0.001	724.33 ± 0.58
	II	3.65 ± 0.036	17.053 ± 0.489	0.270 ± 0.0200	349.0 ± 0.36	1.861 ± 0.001	630.00 ± 1.73
	III	3.75 ± 0.042	17.127 ± 0.239	0.323 ± 0.0493	362.0 ± 0.15	1.886 ± 0.001	682.67 ± 0.58
Mean		*3.75* ± *0.11* ^*a*^	*17.50* ± *0.80* ^*c*^	*0.303* ± *0.04* ^*b*^	*370.01* ± *22.51* ^*c*^	*1.950* ± *0.12* ^*b*^	*679.00* ± *40.95* ^*a*^
*Melipona* sp.	I	3.80 ± 0.027	13.507 ± 0.047	0.217 ± 0.0152	209.0 ± 0.31	1.533 ± 0.000	762.33 ± 2.31
	II	4.18 ± 0.066	15.203 ± 0.186	0.240 ± 0.0100	211.7 ± 0.15	2.012 ± 0.001	792.33 ± 1.15
	III	4.65 ± 0.053	12.863 ± 0.221	0.257 ± 0.0153	221.2 ± 0.15	1.632 ± 0.001	805.33 ± 0.58
Mean		*4.21* ± *0.37* ^*b*^	*13.86* ± *1.06* ^*b*^	*0.238* ± *0.02* ^*a*^	*214.00* ± *5.55* ^*a*^	*1.726* ± *0.22* ^*a*^	*786.67* ± *19.14* ^*b*^

Results were expressed as mean ± standard deviation of duplicate experiments. Means were compared by using One way ANOVA and Games-Howell Post Hoc Multiple Comparisons. In each column, mean values with different letters (superscripts “a–c”) indicate significant differences (*p* < 0.05)

EC, electrical conductivity; TDS, total dissolved solids; Colour (absorbance at 560 nm); ABS_450_, colour intensity. *Hypotrigona* sp. honey—Okotobo honey and *Melipona* sp. honey—Ifufu/Ihuhu honey

There were statistically significant differences between the mean content values of total sugar (F_(2,24)_ = 53.765), reducing sugar (F_(2,24)_ = 35.303), sucrose (F_(2,24)_ = 140.198), (F_(2,24)_ = 250.031), Total (F_(2,24)_ = 6492.145), free (F_(2,24)_ = 3524.638) and lactone (F_(2,24)_ = 123.790) acidities of the honey varieties (Table 2). The honey from *Melipona* sp. had significantly the highest mean values of total sugars (80.71 ± 1.37/100 g), reducing sugars (75.64 ± 1.99/100 g) and sucrose (5.06 ± 0.75%)contents while the *Hypotrigona* sp. honey had significantly the highest mean values of HMF (16.58 ± 0.37 mg/kg), protein content (16.58 ± 0.37 g/kg), total (35.57 ± 0.42 meq/kg), free (30.69 ± 0.32 meq/kg) and lactone (4.88 ± 0.61 meq/kg) acidities (*p* < 0.05) (Table 2).

**Table 2:** Biochemical properties of the honeys

Honey source	Samples	Parameters							
		Total sugar contents [(%) g/g]	Reducing sugar [(%) g/g)]	Sucrose [(%) g/g]	HMF (Mg/kg)	Total protein contents (g/kg)	Total acidity (meq/kg)	Free acidity (meq/kg)	Lactone acidity (meq/kg)
*Apis mellifera*	I	79.61 ± 0.03	77.26 ± 0.03	2.35 ± 0.01	13.17 ± 0.05	3.05 ± 0.05	21.93 ± 0.03	18.31 ± 0.11	3.62 ± 0.09
	II	70.17 ± 0.21	67.75 ± 0.19	2.42 ± 0.02	11.97 ± 0.05	3.11 ± 0.02	22.07 ± 0.10	19.46 ± 0.43	2.94 ± 0.08
	III	68.32 ± 0.01	66.00 ± 0.02	2.32 ± 0.01	16.12 ± 0.12	3.94 ± 0.01	20.96 ± 0.32	18.24 ± 0.25	2.71 ± 0.07
Mean		*72.70* ± *7.5* ^*b*^	*70.34* ± *7.49* ^*b*^	*2.36* ± *0.05* ^*b*^	*13.75* ± *1.85* ^*b*^	*13.75* ± *1.85* ^*b*^	*21.65* ± *0.55* ^*b*^	*18.67* ± *0.64* ^*b*^	*3.09* ± *0.41* ^*b*^
*Hypotrigona* sp.	I	62.72 ± 0.01	60.72 ± 0.02	1.99 ± 0.02	16.39 ± 0.08	6.58 ± 0.03	35.70 ± 0.22	30.85 ± 0.29	4.85 ± 0.10
	II	66.32 ± 0.01	64.64 ± 0.02	1.67 ± 0.02	16.29 ± 0.04	4.99 ± 0.04	35.08 ± 0.30	30.89 ± 0.22	4.19 ± 0.19
	III	57.94 ± 0.03	56.12 ± 0.04	1.82 ± 0.02	17.07 ± 0.05	5.67 ± 0.02	35.93 ± 0.07	30.34 ± 0.03	5.59 ± 0.06
Mean		*62.32* ± *5.25* ^*a*^	*60.49* ± *5.21* ^*a*^	*1.83* ± *0.14* ^*a*^	*16.58* ± *0.37* ^*c*^	*16.58* ± *0.37* ^*c*^	*35.57* ± *0.42* ^*c*^	*30.69* ± *0.32* ^*a*^	*4.88* ± *0.61* ^*c*^
*Melipona* sp.	I	80.53 ± 1.59	75.73 ± 2.25	4.79 ± 0.95	5.50 ± 0.03	2.97 ± 0.04	12.43 ± 0.10	10.63 ± 0.16	1.80 ± 0.07
	II	79.55 ± 0.44	73.72 ± 0.46	5.82 ± 0.02	5.65 ± 0.04	3.94 ± 0.01	12.95 ± 0.07	11.62 ± 0.12	1.32 ± 0.07
	III	82.04 ± 0.19	77.47 ± 0.20	4.57 ± 0.02	5.35 ± 0.12	3.78 ± 0.01	12.40 ± 0.03	11.43 ± 0.05	1.04 ± 0.07
Mean		*80.71* ± *1.37* ^*c*^	*75.64* ± *1.99* ^*c*^	*5.06* ± *0.75* ^*c*^	*5.50* ± *1.15* ^*a*^	*5.50* ± *1.15* ^*a*^	*12.59* ± *0.27* ^*a*^	*11.23* ± *0.47* ^*c*^	*1.39* ± *0.34* ^*a*^

Results were expressed as mean ± standard deviation of duplicate experiments. Means were compared using One way ANOVA and Games-Howell Post Hoc Multiple Comparisons. In each column, mean values with different letters (superscripts “a–c”) indicate significant differences (*p* < 0.05)

HMF, hydroxymethyl furfural. *Hypotrigona* sp. honey—Okotobo honey and *Melipona* sp. honey—Ifufu/Ihuhu honey

A statistically significant differences were observed between the mean values of polyphenol contents (F_(2,24)_ = 154.982), flavonoid contents (F_(2,15)_ = 38.25), proline content (F_(2,24)_ = 14.039), ascorbic acid content (F_(2,24)_ = 13.767), AEAC (F_(2,15)_ = 13.38) and FRAP (F_(2,15)_ = 643.33 (*p* < 0.05) of the investigated honeys (Table 3). The honey samples from *Hypotrigona* sp. had significantly the highest mean value of total phenolic acid content (527.41 ± 3.60 mg _GAE_/kg), proline content (430.17 ± 51.45 mg/kg), ascorbic acid content (161.69 ± 6.70 mg/kg), AEAC (342.33 ± 0.78 mg/kg) and FRAP (666.88 ± 1.73) μM Fe(II)/100 g. Apart from having the highest flavonoid contents, *Melipona* sp. honey had more of these parameters than *Apis mellifera* honey (Table 3).

**Table 3:** Antioxidant properties of the honeys

Honey source	Samples	Parameters					
		Total phenol contents (mg_GAE_/kg)	Flavonoids contents (mg_CEQ_/kg)	Proline contents (mg/kg)	Ascorbic acid contents (mg/kg)	AEAC contents (mg/kg)	FRAP (µM Fe(II)/100 g
*Apis mellifera*	I	409.73 ± 1.95	57.00 ± 0.16	338.89 ± 0.13	157.44 ± 0.49	304.51 ± 0.16	525.68 ± 0.83
	II	432.07 ± 0.10	62.35 ± 1.09	339.21 ± 0.01	161.96 ± 0.05	299.22 ± 8.44	508.45 ± 17.51
	III	475.68 ± 0.41	65.82 ± 0.29	481.27 ± 0.03	149.48 ± 0.28	290.67 ± 0.58	500.06 ± 0.05
Mean		*439.16* ± *29.06* ^*b*^	*61.72* ± *3.89* ^*b*^	*386.46* ± *71.11* ^*b*^	*156.29* ± *5.48* ^*b*^	*298.13* ± *7.38* ^*a*^	*511.40* ± *14.31* ^*b*^
*Hypotrigona* sp.	I	531.10 ± 0.05	52.52 ± 0.03	498.52 ± 0.04	169.27 ± 0.04	343.04 ± 0.89	668.53 ± 0.23
	II	522.91 ± 0.10	28.19 ± 0.03	401.26 ± 0.26	153.80 ± 0.07	342.34 ± 0.29	667.24 ± 1.22
	III	375.82 ± 0.25	43.41 ± 0.22	390.73 ± 0.25	162.01 ± 0.23	341.62 ± 0.92	664.87 ± 0.22
Mean		*527.41* ± *3.60* ^*c*^	*41.37* ± *10.65* ^*a*^	*430.17* ± *51.45* ^*b*^	*161.69* ± *6.70* ^*c*^	*342.33* ± *0.78* ^*b*^	*666.88* ± *1.73* ^*c*^
*Melipona* sp.	I	386.09 ± 0.11	82.78 ± 0.39	281.28 ± 0.06	149.69 ± 0.27	293.44 ± 0.49	439.15 ± 0.34
	II	354.03 ± 0.08	83.79 ± 0.06	276.11 ± 0.03	151.62 ± 0.34	297.49 ± 3.77	424.28 ± 12.69
	III	375.82 ± 0.25	92.60 ± 0.10	338.84 ± 0.07	144.31 ± 0.08	299.77 ± 1.35	417.36 ± 0.52
Mean		*371.98* ± *14.18* ^*a*^	*86.39* ± *4.69* ^*c*^	*298.74* ± *3016* ^*a*^	*148.54* ± *3.29* ^*a*^	*296.99* ± *3.43* ^*a*^	*426.93* ± *11.55* ^*a*^

Results were expressed as mean ± standard deviation of duplicate experiments. Means were compared by using One way ANOVA and Games-Howell Post Hoc Multiple Comparisons. In each column, mean values with different letters (superscripts “a–c”) indicate significant differences (*p* < 0.05)

GAE, gallic acid equivalent; CEQ, catechin equivalent; FRAP, ferric reducing/antioxidant power assay; AEAC, antioxidant equivalent—ascorbic acid assay. *Hypotrigona* sp. honey—Okotobo honey and *Melipona* sp. honey—Ifufu/Ihuhu honey

### Correlation among some physicochemical and antioxidant parameters

The correlation matrixes showed significant correlations between some of the physicochemical and antioxidant parameters. In *A. mellifera* honey samples, a strong correlation was found between colour intensity and some parameters (Additional file 1: Table S1). Also, correlation matrixes of *Hypotrigona* sp. honey samples showed strong correlations between ABS_450_ and phenolics flavonoid, protein, ascorbic acid and AEAC. The proline content had positive correlations with total phenol content, AEAC, FRAP and flavonoid content (Additional file 1: Table S2). Several strong positive correlations were established between ABS_450_ of *Melipona* sp. and protein and AEAC. The flavonoid contents had correlation with proline, AAC, AEAC and FRAP (Additional file 1: Table S3).

## Discussion

All honey varieties analysed were acidic. The observed pH values in this study are comparatively similar to previous reports for Nigerian stinging bee honey (3.32 and 4.90) [6, 9] and higher than stingless bee honeys (1.40–3.97) [10]. None of the investigated samples exceeded the allowed limit of international standards (pH of 3.42–6.10) [25]. The moisture content of the honey samples are within the recommended international standard limit (not more than 20%) [25, 26]. The moisture content of the tested honeys may be related to the harvest season and the level of maturity in the hive [11]. While similar findings have been reported by Omoya et al. [7] and Eleazu et al. [9], and a higher moisture content values (25.43–26.51%) has been reported for Nigeria stingless bee honey [10]. *Hypotrigona* sp. honey showed the highest mean EC value and the highest mean content of TDS, which indicates that it is rich in both organic and inorganic substances [12]. The EC values of the investigated honey samples are within the allowed limit of international standards (not more than 0.8 mSs/cm) [25]. This is the first report on ABS_450_ of Nigeria honeys and similarly, the ABS_450_ values for Slovenian, Algerian and Indian honeys were reported to be 70–495 mAU [14], 724–1188 mAU [27], and 524–1678 mAU [16]), respectively. Comparatively, a smaller strong correlation was found between the colour intensity of Malaysian [28] and Algerian [27] honey samples and the other antioxidant parameters, like flavonoid.

In this study, *Hypotrigona* sp. and *Melipona* sp. honeys had the highest and lowest mean total sugar content value, respectively. The sugars contents values are within the limits (Reducing sugar—not less than 60% (g/100 g) and sucrose—not more than 5% [g/100 g)] set by the Codex Alimentarius [25] and European Commission [26]. *Hypotrigona* sp. had the highest mean HMF value and our results were within the recommended standard set by Codex Alimentarius [25] and EU [26] (maximum of 40 mg/kg). These findings are similar to other published levels for HMF on Nigerian honeys (5.0–17.22 mg/kg) [8], Algerian honeys (15.23–24.21 mg/kg) [27] and Pakistan honeys (27.69–36.08 mg/kg) [29]. The low HMF concentrations confirmed that these samples are of good quality. The free acidity values of the analysed honey samples are within the international standard (not more than 50 meq/kg) [25].

The total polyphenol content of analysed *A. mellifera* honey samples are lower than previous report on Nigerian honey samples (846.00–1087.00 mg _GAE_/kg) [9] and comparatively similar to Algerian (411.10–498.16 mg _GAE_/kg) [27] and Malaysian (144.51–580.03 mg _GAE_/kg) [28] honeys. There was no strong positive correlation between total polyphenol content and AEAC or FRAP, although, a weak positive correlations have been reported for Algerian honey [27]. *Melipona* sp. honey showed the highest flavonoid contents values, followed by *A. mellifera* honey, similar to Algerian honey (27.07–71.78 mg _CEQ_/kg) [27] and Turkish honeys (28.17–87.01 mg _CEQ_/kg) [3]. Malaysian honey has been reported to have a correlation (0.782) between flavonoid and FRAP [28], unlike observed in this study. In this study, the stingless bee honeys showed higher ascorbic acid content values than previously reported for Nigerian stingless bee honey (119.5–120.0 mg/kg) [10] and Portugal (140–145 mg/kg) [22]. The AEAC content values observed in this study are within the range of values reported for honeys from India (151–295 mg of AEAC/kg) [16] and Algeria (236.80–315.90 mg of AEAC/kg) [27]. All honey varieties investigated showed high FRAP values, confirming its high antioxidant properties and are similar to that reported for Malaysian honeys (209.28–653.75 μM Fe(II)/100 g) [28] and Italian honey (216.57–695.64 μM Fe(II)/100 g) [30], but higher than Algerian honeys (287.45–403.54 μM Fe(II)/100 g) [27].

## Conclusion

Our results clearly indicate that *Hypotrigona* sp. honey possesses the best antioxidant property when compared with honeys from *A. mellifera* and *Melipona* sp. *Hypotrigona* sp. (Okotobo) and *Melipona* sp. (Ifufu) honeys are not widely consumed as regular bee honey but this study has shown that they contain bioactive compounds similar to those of regular bee honey.

### Limitations

This study was performed using some of the standard analytical procedures and it has main limitations in terms of the number of honey samples and methods used, which may have resulted in underestimation of the parameters. A more precise equipment such as HPLC should in future be used to determine these parameters.

## Additional file

**Additional file 1.** Interrelation among some physicochemical and antioxidant parameters of different honey types.

## Abbreviations

EC: electrical conductivity
TDS: total dissolved solids
HMF: hydroxymethylfurfural
ABS_450_: colour intensity
AEAC: antioxidant equivalent—ascorbic acid content
FRAP: ferric reducing/antioxidant power
DNSA: 3,5-dinitrosalicylic acid
CEQ: catechin equivalents
GAE: gallic acid equivalents
ANOVA: analysis of variance
