# Supplementary material for: Evaluation of physicochemical and antioxidant properties of two stingless bee honeys: a comparison with Apis mellifera honey from Nsukka, Nigeria
Source: BMC Res Notes. 2017 Nov 6;10:566. doi: 10.1186/s13104-017-2884-2 (PMC5674770; doi:10.1186/s13104-017-2884-2)
Supplement: Supplementary file 1 — Additional file 1. Interrelation among some physicochemical and antioxidant parameters of different honey types. [file 13104_2017_2884_MOESM1_ESM.docx]

**ADDITIONAL 1 (TABLES A, B and C)**

**Table S1:** Correlation matrix showing the interrelation among some physicochemical and antioxidant parameters of *Apis mellifera* honey

|  | ABS_450_ | EC | Protein | TPC | FC | Proline | AAC | AEAC | FRAP |
| --- | --- | --- | --- | --- | --- | --- | --- | --- | --- |
| ABS_450_ | 1.000 | 0.975^**^ | 0.833^**^ | 0.954^**^ | 0.990^**^ | 0.803^**^ | -0.536 | -0.800^**^ | -0.789^*^ |
| EC | 0.975^**^ | 1.000 | 0.699^*^ | 0.857^**^ | 0.899^**^ | 0.682^*^ | -0.397 | -0.678^*^ | 0.727^*^ |
| Protein | 0.833^**^ | 0.699^*^ | 1.000 | 0.957^**^ | 0.821^**^ | 0.996^**^ | -0.911^**^ | -0.773^*^ | -0.615 |
| TPC | 0.954^**^ | 0.857^**^ | 0.957^**^ | 1.000 | 0.943^**^ | 0.943^**^ | -0.759^*^ | -0.860^**^ | -0.733^*^ |
| TFC | 0.990^**^ | 0.899^**^ | 0.821^**^ | 0.943^**^ | 1.000 | 0.792^*^ | -0.525 | -0.860^**^ | -0.865^**^ |
| Proline | 0.803^**^ | 0.682^*^ | 0.996^**^ | 0.943^**^ | 0.792^*^ | 1.000 | -0.932^**^ | -0.759^*^ | -0.595 |
| AAC | -0.536 | -0.397 | -0.911^**^ | -0.759^*^ | -0.525 | -0.932^**^ | 1.000 | 0.595 | 0.366 |
| AEAC | -0.800^**^ | -0.678^*^ | -0.773^*^ | -0.860^**^ | -0.860^**^ | -0.759^*^ | 0.595 | 1.000 | 0.930^**^ |
| FRAP | -0.789^*^ | 0.727^*^ | -0.615 | -0.733^*^ | -0.865^**^ | -0.595 | 0.366 | 0.930^**^ | 1.000 |
| **. Correlation is significant at the 0.01 level (2-tailed); *. Correlation is significant at the 0.05 level (2-tailed). ABS_450_ – Colour intensity (µAU,50w/v), EC – Electrical conductivity (mS/cm), Protein content (g/kg), TPC - Total phenol content (mg GAE/kg), FC - Flavonoid content (mg CEQ/kg), Proline content (mg/kg), AAC - Ascorbic acid content (mg/kg), AEAC - Antioxidant equivalent - ascorbic acid contents (mg/kg), FRAP - Ferric reducing/antioxidant power (µM Fe(II)/100g | | | | | | | | | |

**Table S2:** Correlation matrix showing the interrelation among some physicochemical and antioxidant parameters of *Hypotrigona* sp. honey

|  | ABS_450_ | EC | Protein | TPC | FC | Proline | AAC | AEAC | FRAP |
| --- | --- | --- | --- | --- | --- | --- | --- | --- | --- |
| ABS_450_ | 1.000 | 0.574 | 0.987^**^ | 0.995^**^ | 0.997^**^ | 0.778^*^ | 0.999^**^ | 0.345 | 0.260 |
| EC | 0.574 | 1.000 | 0.512 | 0.613 | 0.603 | 0.216 | 0.567 | -0.119 | -0.218 |
| Protein | 0.987^**^ | 0.512 | 1.000 | 0.967^**^ | 0.973^**^ | 0.864^**^ | 0.992^**^ | 0.444 | 0.404 |
| TPC | 0.995^**^ | 0.613 | 0.967^**^ | 1.000 | 1.000^**^ | 0.710^*^ | 0.991^**^ | 0.271 | 0.166 |
| TFC | 0.997^**^ | 0.603 | 0.973^**^ | 1.000^**^ | 1.000 | 0.727^*^ | 0.994^**^ | 0.288 | 0.192 |
| Proline | 0.778^*^ | 0.216 | 0.864^**^ | 0.710^*^ | 0.727^*^ | 1.000 | 0.797^*^ | 0.717^*^ | 0.766* |
| AAC | 0.999^**^ | 0.567 | 0.992^**^ | 0.991^**^ | 0.994^**^ | 0.797^*^ | 1.000 | 0.368 | 0.293 |
| AEAC | 0.345 | -0.119 | 0.444 | 0.271 | 0.288 | 0.717^*^ | 0.368 | 1.000 | 0.769^*^ |
| FRAP | 0.26 | -0.218 | 0.404 | 0.166 | 0.192 | 0.766* | 0.293 | 0.769^*^ | 1.000 |
| **. Correlation is significant at the 0.01 level (2-tailed); *. Correlation is significant at the 0.05 level (2-tailed). ABS_450_ – Colour intensity (µAU,50w/v), EC – Electrical conductivity (mS/cm), Protein content (g/kg), TPC - Total phenol content (mg GAE/kg), FC - Flavonoid content (mg CEQ/kg), Proline content (mg/kg), AAC - Ascorbic acid content (mg/kg), AEAC - Antioxidant equivalent - ascorbic acid contents (mg/kg), FRAP - Ferric reducing/antioxidant power (µM Fe(II)/100g | | | | | | | | | |

**Table S3:** Correlation matrix showing the interrelation among some physicochemical and antioxidant parameters of from *Melipona* sp. honey

|  | ABS_450_ | EC | Protein | TPC | FC | Proline | AAC | AEAC | FRAP |
| --- | --- | --- | --- | --- | --- | --- | --- | --- | --- |
| ABS_450_ | 1.000 | 0.834^**^ | 0.898^**^ | -0.515 | 0.792^*^ | 0.679^*^ | -0.530 | 0.826^**^ | -0.850 |
| EC |  | 1.000 |  |  |  |  |  |  |  |
| Protein | 0.898^**^ | 0.697^*^ | 1.000 | -0.839^**^ | 0.446 | 0.290 | -0.109 | 0.702^*^ | -0.742 |
| TPC | -0.515 | -0.331 | -0.839^**^ | 1.000 | 0.111 | 0.275 | -0.445 | -0.373 | 0.419 |
| TFC | 0.792^*^ | 0.712^**^ | 0.446 | 0.111 | 1.000 | 0.985^**^ | -0.935^**^ | 0.675^*^ | -0.674^*^ |
| Proline | 0.679^*^ | 0.635 | 0.290 | 0.275 | 0.985^**^ | 1.000 | -0.981^**^ | 0.587 | -0.579 |
| AAC | -0.530 | -0.502 | -0.109 | -0.445 | -0.935^**^ | -0.981^**^ | 1.000 | -0.461 | 0.442 |
| AEAC | 0.826^**^ | 0.593 | 0.702^*^ | -0.373 | 0.675^*^ | 0.587 | -0.461 | 1.000 | -0.975^**^ |
| FRAP | -0.850 | -0.677^*^ | -0.742 | 0.419 | -0.674^*^ | -0.579 | 0.442 | -0.975^**^ | 1.000 |
| **. Correlation is significant at the 0.01 level (2-tailed); *. Correlation is significant at the 0.05 level (2-tailed). ABS_450_ – Colour intensity (µAU,50w/v), EC – Electrical conductivity (mS/cm), Protein content (g/kg), TPC - Total phenol content (mg GAE/kg), FC - flavonoid content (mg CEQ/kg), Proline content (mg/kg), AAC - Ascorbic acid content (mg/kg), AEAC - Antioxidant equivalent - ascorbic acid contents (mg/kg), FRAP - Ferric reducing/antioxidant power (µM Fe(II)/100g | | | | | | | | | |
